# Supplementary material for: Nirmatrelvir-resistant SARS-CoV-2 is efficiently transmitted in female Syrian hamsters and retains partial susceptibility to treatment
Source: Nat Commun. 2023 Apr 14;14:2124. doi: 10.1038/s41467-023-37773-6 (PMC10101821; doi:10.1038/s41467-023-37773-6)
Supplement: Supplementary file 1 — Supplementary Information [file 41467_2023_37773_MOESM1_ESM.pdf]

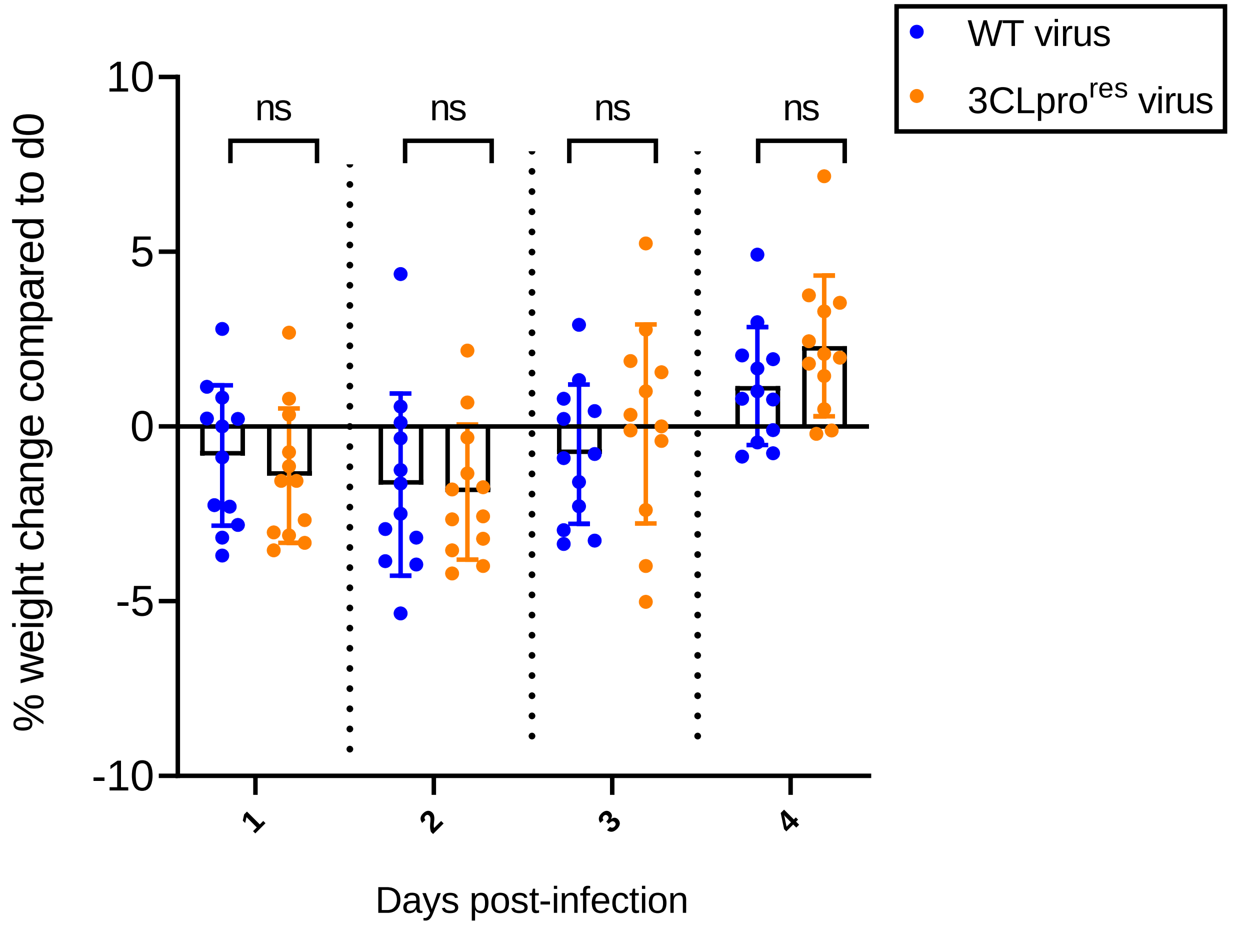

Fig. S1. Body weight change of Syrian hamsters infected with either the WT or 3CLpro resistant virus. Weight change at days 1-4 post-infection in percentage, normalized to the body weight at the time of infection (day zero, d0). Bars represent means ± SD. Data were analyzed with the two-sided Mann−Whitney U test. Ns= non-significant ($p>0.05$). Data presented are from 2-independent studies with a total n=12 per group.

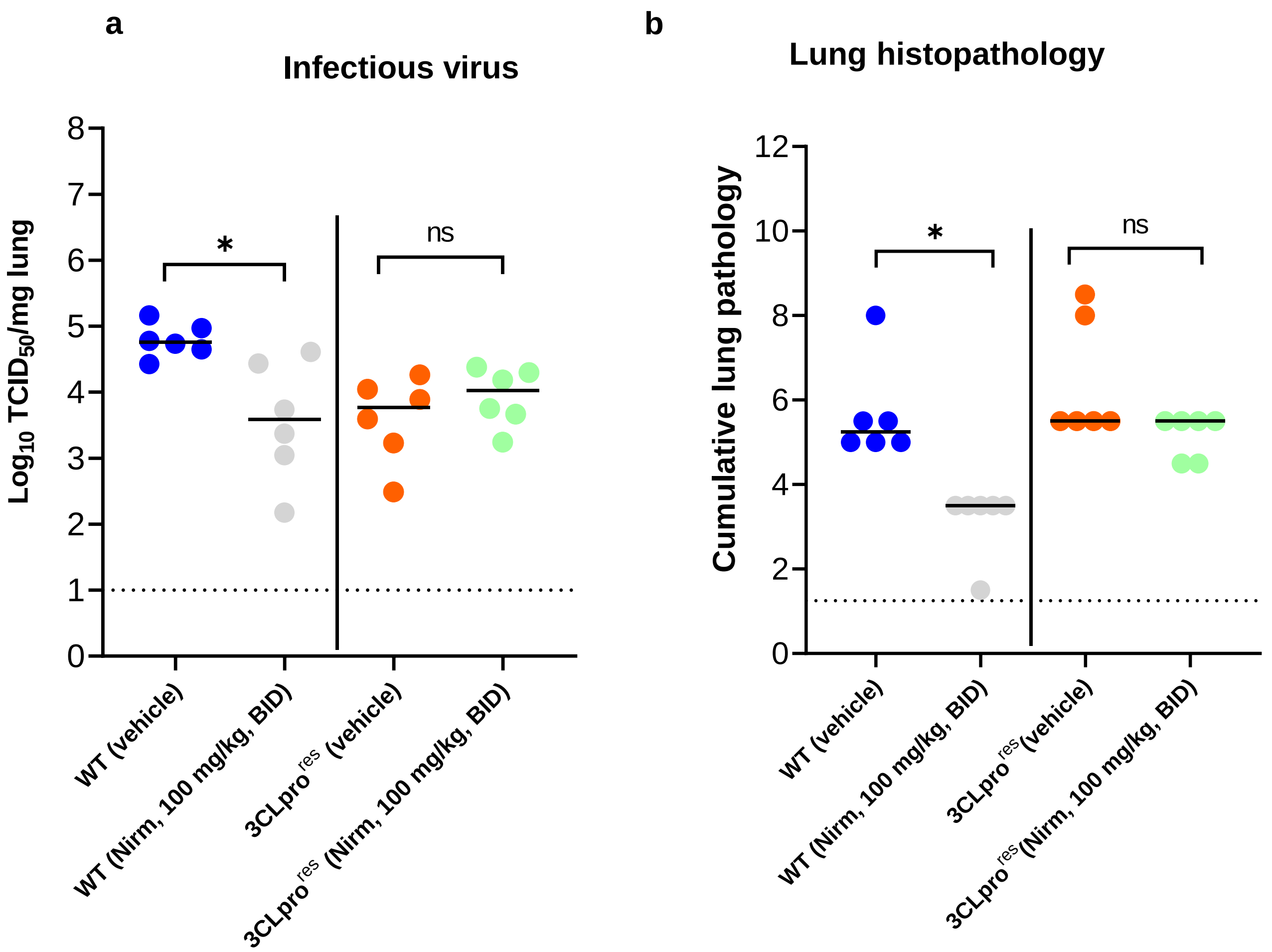

Fig. S2. *In vivo* efficacy of nirmatrelvir at 100 mg/kg (BID) against the 3CLpro (L50F-E166A-L167F) resistant virus. (a) Infectious viral loads in the lungs of hamsters that were treated with vehicle or nirmatrelvir (Nirm) at 100 mg/kg (BID) and infected with 10^4 $TCID_{50}$ of either the wild-type (WT) SARS-CoV-2 isolate (USA-WA1/2020) or the 3CLpro (L50F-E166A-L167F) nirmatrelvir resistant ($3CLpro^{res}$) virus at day 4 post-infection (pi) are expressed as log_{10} $TCID_{50}$ per mg lung tissue. Individual data and median values are presented. b) Cumulative severity score at day 4 p.i. from H&E stained slides of lungs from hamsters treated with either vehicle or nirmatrelvir (Nirm) and infected with either the WT SARS-CoV-2 isolate or $3CLpro^{res}$ virus. Individual data and median values are presented; the dotted line represents the median score of untreated non-infected hamsters. Data were analyzed with the Kruskal-Wallis test, *p<0.022 and 0.035 for panel a and b, respectively, ns=non-significant. Data presented are from a single study with n=6 per group.

A) WT virus

B) 3CLpro^{res} virus

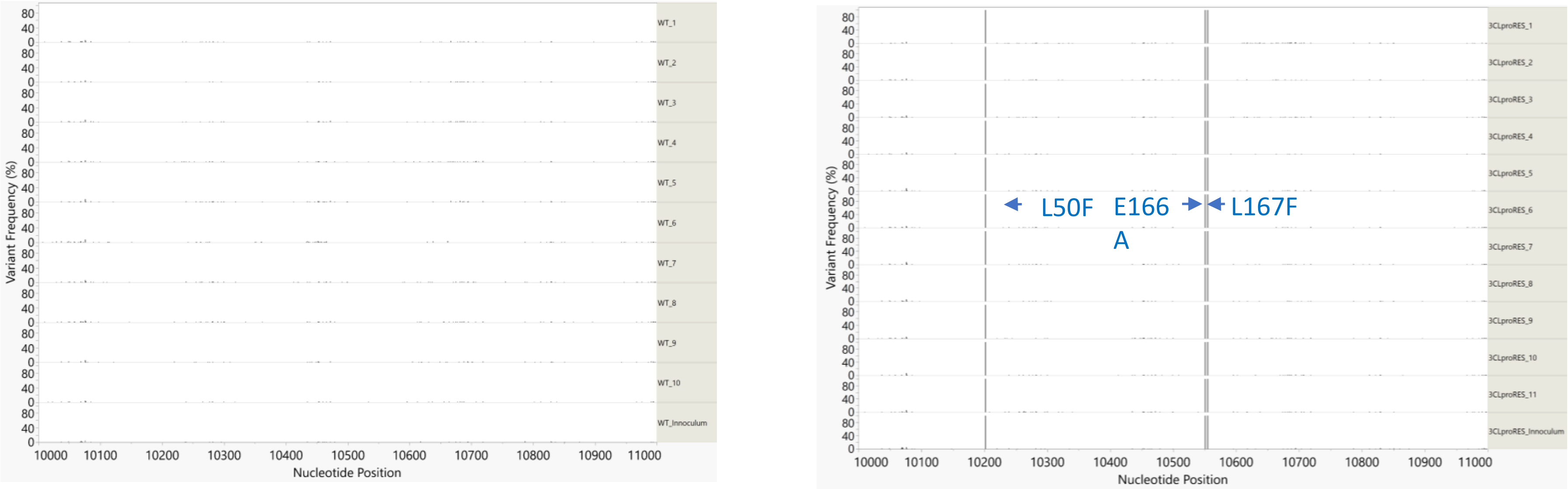

Fig. S3. Alignment of SARS-CoV-2 3CLpro gene sequences obtained by deep sequencing of the viral RNA from the lungs of niramterlvir-treated hamsters that were infected with either A) WT or B) the 3CLpro^{res} virus.

A) WT virus

B) 3CLpro^{res} virus

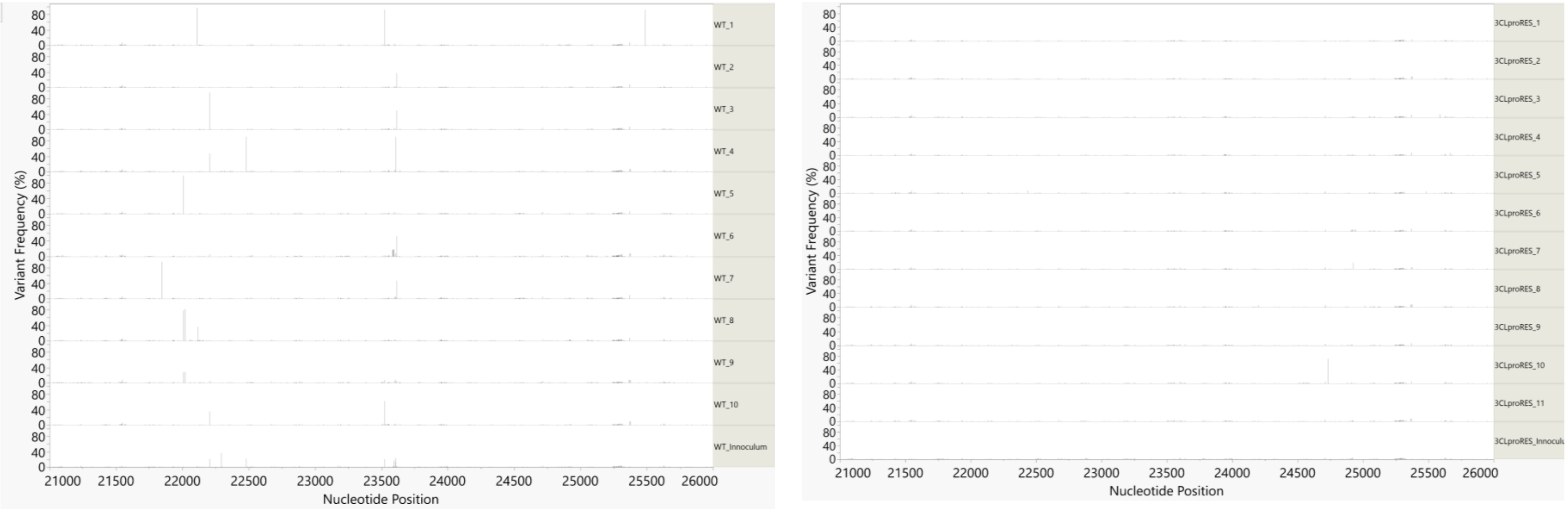

Fig. S4. Alignment of SARS-CoV-2 spike gene sequences obtained by deep sequencing of the viral RNA from the lungs of niramterlvir-treated hamsters that were infected with either A) WT or B) the 3CLpro^{res} virus.
