# Supplementary material for: Nirmatrelvir-resistant SARS-CoV-2 is efficiently transmitted in female Syrian hamsters and retains partial susceptibility to treatment
Source: Nat Commun. 2023 Apr 14;14:2124. doi: 10.1038/s41467-023-37773-6 (PMC10101821; doi:10.1038/s41467-023-37773-6)
Supplement: Supplementary file 3 — Reporting Summary [file 41467_2023_37773_MOESM3_ESM.pdf]

nature portfolio

Corresponding author(s): Johan Neyts, NCOMMS-22-45423

Last updated by author(s): Nov 16, 2022

Reporting Summary

Nature Portfolio wishes to improve the reproducibility of the work that we publish. This form provides structure for consistency and transparency in reporting. For further information on Nature Portfolio policies, see our Editorial Policies and the Editorial Policy Checklist.

Statistics

For all statistical analyses, confirm that the following items are present in the figure legend, table legend, main text, or Methods section.

n/a	Confirmed	
☐	☒	The exact sample size (*n*) for each experimental group/condition, given as a discrete number and unit of measurement
☐	☒	A statement on whether measurements were taken from distinct samples or whether the same sample was measured repeatedly
☐	☒	The statistical test(s) used AND whether they are one- or two-sided<br>*Only common tests should be described solely by name; describe more complex techniques in the Methods section.*
☒	☐	A description of all covariates tested
☒	☐	A description of any assumptions or corrections, such as tests of normality and adjustment for multiple comparisons
☐	☒	A full description of the statistical parameters including central tendency (e.g. means) or other basic estimates (e.g. regression coefficient) AND variation (e.g. standard deviation) or associated estimates of uncertainty (e.g. confidence intervals)
☐	☒	For null hypothesis testing, the test statistic (e.g. *F*, *t*, *r*) with confidence intervals, effect sizes, degrees of freedom and *P* value noted<br>*Give P values as exact values whenever suitable.*
☒	☐	For Bayesian analysis, information on the choice of priors and Markov chain Monte Carlo settings
☒	☐	For hierarchical and complex designs, identification of the appropriate level for tests and full reporting of outcomes
☐	☒	Estimates of effect sizes (e.g. Cohen's *d*, Pearson's *r*), indicating how they were calculated

Our web collection on statistics for biologists contains articles on many of the points above.

Software and code

Policy information about availability of computer code

Data collection	No software was used
Data analysis	All statistical analyses were performed using GraphPad Prism 9 software (GraphPad, San Diego, CA, USA), Alignment of gene sequences was performed using the Geneious Prime software.

For manuscripts utilizing custom algorithms or software that are central to the research but not yet described in published literature, software must be made available to editors and reviewers. We strongly encourage code deposition in a community repository (e.g. GitHub). See the Nature Portfolio guidelines for submitting code & software for further information.

Data

Policy information about availability of data

All manuscripts must include a data availability statement. This statement should provide the following information, where applicable:

- Accession codes, unique identifiers, or web links for publicly available datasets
- A description of any restrictions on data availability
- For clinical datasets or third party data, please ensure that the statement adheres to our policy

All of the data generated or analysed during this study are included in this published article. The deep sequencing data of the viral RNA from the transmission study are available on SRA under BioProject: PRJNA925007 with accession numbers SRX19069060-SRX19069082 and SRX19058198. All data supporting the findings in this study are also available from the corresponding author upon request.

Field-specific reporting

Please select the one below that is the best fit for your research. If you are not sure, read the appropriate sections before making your selection.

☒ Life sciences ☐ Behavioural & social sciences ☐ Ecological, evolutionary & environmental sciences

For a reference copy of the document with all sections, see nature.com/documents/nr-reporting-summary-flat.pdf

Life sciences study design

All studies must disclose on these points even when the disclosure is negative.

Sample size	For in vivo antiviral efficacy, we want to detect at least 1 log10 reduction in viral RNA levels in treated subjects compared to the untreated, infected control group. Group size was calculated on the independent t-test with an effect size of 2.0 and a power of 80% (effect size = deltamean/SD = 1 log10 decrease in viral RNA/0.5 log10), resulting in 5-6 animals/group. Sample sizes maximized considering limits in BSL3 housing capacity, numbers of animals that can be handled under BSL3 conditions, and availability of compounds.
Data exclusions	No data exclusions
Replication	two independent studies were done
Randomization	No randomization methods were used and confounders were not controlled. Since all animals had the same age and roughly of the same weight; all are females, we believe there is no a real confounder to be considered in the study that may impact the outcome of the experiments.
Blinding	All caretakers and technicians were blinded to group allocation in the animal facility. In addition analysis of data (qPCR, titration and histology) was done blindly without revealing of groups ID at the time of analysis

Reporting for specific materials, systems and methods

We require information from authors about some types of materials, experimental systems and methods used in many studies. Here, indicate whether each material, system or method listed is relevant to your study. If you are not sure if a list item applies to your research, read the appropriate section before selecting a response.

Materials & experimental systems

n/a	Involved in the study	
☒	☐	Antibodies
☐	☒	Eukaryotic cell lines
☒	☐	Palaeontology and archaeology
☐	☒	Animals and other organisms
☒	☐	Human research participants
☒	☐	Clinical data
☒	☐	Dual use research of concern

Methods

n/a	Involved in the study	
☒	☐	ChIP-seq
☒	☐	Flow cytometry
☒	☐	MRI-based neuroimaging

Eukaryotic cell lines

Policy information about cell lines

Cell line source(s)	Vero E6 cells (African green monkey kidney, ATCC CRL-1586)
Authentication	No authentication was performed
Mycoplasma contamination	We confirm that all cells were tested negative for Mycoplasma
Commonly misidentified lines (See ICLAC register)	not applicable

Animals and other organisms

Policy information about studies involving animals; ARRIVE guidelines recommended for reporting animal research

Laboratory animals	Female Syrian hamsters (Mesocricetus auratus) were purchased from Janvier Laboratories and kept per two in individually ventilated isolator cages (IsoCage N Bio-containment System, Tecniplast)

Wild animals	Study did not invlove wild animals
Field-collected samples	The study did involve field-collected samples
Ethics oversight	Housing conditions and experimental procedures were done with the approval and under the guidelines of the ethics committee of animal experimentation of KU Leuven (license P065-2020).

Note that full information on the approval of the study protocol must also be provided in the manuscript.
